# Immune mechanisms in epileptogenesis

**DOI:** 10.3389/fncel.2013.00195

**Published:** 2013-11-08

**Authors:** Dan Xu, Stephen D. Miller, Sookyong Koh

**Affiliations:** ^1^Department of Microbiology-Immunology and Interdepartmental Immunobiology, Feinberg School of Medicine, Northwestern UniversityChicago IL, USA; ^2^Department of Pediatrics, Division of Neurobiology, Children’s Research Center, Lurie Children’s Hospital of ChicagoChicago IL, USA; ^3^Department of Pediatrics, Feinberg School of Medicine, Northwestern UniversityChicago IL, USA

**Keywords:** seizure, epilepsy, epileptogenesis, immune response, inflammation, microglia, astrocytes, T lymphocytes

## Abstract

Epilepsy is a chronic brain disorder that affects 1% of the human population worldwide. Immune responses are implicated in seizure induction and the development of epilepsy. Pre-clinical and clinical evidence have accumulated to suggest a positive feedback cycle between brain inflammation and epileptogenesis. Prolonged or recurrent seizures and brain injuries lead to upregulation of proinflammatory cytokines and activated immune responses to further increase seizure susceptibility, promote neuronal excitability, and induce blood–brain barrier breakdown. This review focuses on the potential role of innate and adaptive immune responses in the pathogenesis of epilepsy. Both human studies and animal models that help delineate the contributions of brain inflammation in epileptogenesis will be discussed. We highlight the critical role of brain-resident immune mediators and emphasize the contribution of brain-infiltrating peripheral leukocytes. Additionally, we propose possible immune mechanisms that underlie epileptogenesis. Several proinflammatory pathways are discussed, including the interleukin-1 receptor/toll-like receptor signaling cascade, the pathways activated by damage-associated molecular patterns, and the cyclooxygenase-2/prostaglandin pathway. Finally, development of better therapies that target the key constituents and processes identified in these mechanisms are considered, for instance, engineering antagonizing agents that effectively block these pathways in an antigen-specific manner.

## INTRODUCTION

Epilepsy is a chronic neurological condition characterized by recurring seizures, and is often accompanied by cognitive deficits and mood disorder (Devinsky, 2004; Pellock, 2004; Jones et al., 2008). It affects approximately 1% of the world population, thus represents one of the most common brain disorders. Epilepsy arises from diverse etiologies including genetic, structural, metabolic, or in other instances, the cause is unknown. There is currently no medication available to effectively prevent epilepsy by targeting the mechanisms underlying the enduring predisposition to recurrent seizures, and nearly half of the patients with epilepsy fail to respond to anticonvulsants that only alleviate symptoms. Thus, there is a pressing need for the development of effective disease-modifying therapies that treat the underlying pathology. Such development can best be accomplished through an in depth understanding of the disease mechanisms.

Until a decade ago, epilepsy research focused on alterations of neuronal activities. Such neurocentric emphasis failed to address questions that arose in more complex models of epileptogenesis. A cumulative body of knowledge has suggested that the pathogenesis of epilepsy is associated with non-neuronal components, such as the glial cells that exceedingly outnumber neurons, brain vascular cells, and more importantly leukocytes from the periphery. Despite a long-held belief that the brain is an immunoprivileged site due to the vascular blood–brain barrier (BBB) that tightly regulates infiltration of blood constituents and the lack of a lymphatic drainage, mounting evidence has supported the critical role of immune responses in the initiation and maintenance of epilepsy (Vezzani and Granata, 2005; Vezzani and Baram, 2007; Choi et al., 2009; Riazi et al., 2010). Ongoing brain inflammation has the potential to lower seizure threshold, which in turn may promote neuronal excitability through modifications of neuronal channels, alterations of neurotransmitter uptake or release, and regulation of BBB permeability (Viviani et al., 2007; Wetherington et al., 2008; Friedman et al., 2009; Vezzani et al., 2011a).

Both innate and adaptive immune responses can be primed in the brain with the contribution of resident immune cells and mediators, as well as leukocytes infiltrating from the periphery (Ransohoff et al., 2003; Banks and Erickson, 2010). The innate arm of the response involves the activation of the IL-1 receptor/toll-like receptor (IL-1R/TLR) signaling pathways through ligation of pathogen-associated molecular patterns (PAMPs) or damage-associated molecular patterns (DAMPs), activation of the cyclooxygenase-2 (COX-2) pathway, and initiation of the transforming growth factor-β/small mothers against decapentaplegic (TGF-β/Smad) signaling cascade. Inflammatory mediators produced by the innate immune system remodel the BBB by enhancing its permeability and upregulating leukocyte adhesion molecules on the endothelium, which acts to attract lymphocytes of the adaptive immune system leading to their infiltration into the CNS.

This review focuses on the roles of immune responses in the pathogenesis of epilepsy by summarizing the most recent findings generated from human studies and animal models that help delineate the contributions of brain inflammation in epileptogenesis. We will evaluate the causal relationship between inflammation and seizure activities and the positive feedback loop they establish by revisiting the experimental evidence from *in vivo* and *in vitro* models. Furthermore, we will provide mechanistic insights into the immunological cascade that precedes the establishment of epilepsy and assess the influences of immune mediators apart from the neurological aspect of seizure induction. Finally, we will propose potential mechanisms that underlie epileptogenesis and discuss development of therapies targeting the key constituents and processes identified in these mechanisms.

## PRE-CLINICAL DATA UNDERSCORING THE RELEVANCE OF IMMUNE RESPONSES IN EPILEPSY

Rodent models of epilepsy have provided ample evidence supporting the role of immune responses in the precipitation of epilepsy, modulation of seizure threshold, orchestration of seizure recurrence, regulation of brain cell survival or attrition, and rewiring of neuronal circuits that may lead to establishment of hyperexcitable neuronal networks (Dube et al., 2005; Kulkarni and Dhir, 2009; Riazi et al., 2010; Vezzani et al., 2011a,b). Adult and immature rats and mice are frequently used to elucidate the role of various immunological pathways that are potentially involved in seizure generation. Administration of proinflammatory or anti-inflammatory agents to rats and mice has been used to assess the influence of these immune mediators on latency to onset, frequency, duration, and phenotype of provoked seizures. Furthermore, the inflammatory pathways can be blocked pharmacologically in wildtype animals or manipulated in transgenic mice to evaluate their role in seizure severity (Kulkarni and Dhir, 2009; Maroso et al., 2010; Vezzani et al., 2011a). Additionally, the availability of genetically modified mice with impaired or constitutively hyperactive immunoregulatory pathways enables more detailed mechanistic studies of inflammation-related epileptogenesis (Campbell et al., 1993; Probert et al., 1997). Guinea pigs models, though less common, have also been used to elucidate the contribution of peripheral immune cells to seizure induction (Librizzi et al., 2010).

### RESIDENT IMMUNE MEDIATORS OF THE BRAIN

It has long been appreciated that chemically induced or electrically stimulated prolonged seizures in rats and mice lead to induction of robust immune responses in the seizure-laden brain (Minami et al., 1991; Eriksson et al., 2000; De Simoni et al., 2000; Vezzani et al., 2000; Turrin and Rivest, 2004; Voutsinos-Porche et al., 2004; Gorter et al., 2006; Jung et al., 2006; Yoshikawa et al., 2006; Aronica et al., 2007; Dhote et al., 2007; Lee et al., 2007; Kulkarni and Dhir, 2009; Holtman et al., 2010; Polascheck et al., 2010). Immunohistochemical analyses performed on sections of affected brains have revealed that various cell type, including microglia, astrocytes, neurons, ependymal cells in the ventricles, and endothelial cells of the BBB, were involved in the waves of inflammation associated with seizure induction (Takao et al., 1990; Ban et al., 1991; Turrin and Rivest, 2004; Chakravarty and Herkenham, 2005; Ravizza and Vezzani, 2006). Activation of the IL-1R/TLR inflammatory pathway was among the first to be identified in these brain resident immune cells. Constitutive expression of IL-1R and TLR has been detected in the brain, though at a suboptimal level. Upon stimulation, such as viral or bacterial infections, cellular injuries, ischemia, and seizures, upregulation of these receptors is readily detectable (Ericsson et al., 1995; Allan et al., 2005; Peltier et al., 2010; Zurolo et al., 2011). Activation of TLRs, particularly TLR2 and TLR4, through systemic or cortical delivery of lipopolysaccharide (LPS) to rats, results in rapid changes in neuronal excitability, e.g., alteration in synaptic transmission and regulation of long-term potentiation (Bellinger et al., 1993; Plata-Salaman and Ffrench-Mullen, 1994; Schneider et al., 1998; Rodgers et al., 2009). The excitatory effect of IL-1β has also been reported to be associated with the reduction of gamma-aminobutyric acid (GABA) inhibition and reduced outward current of voltage-gated Ca^2^^+^ channels in the hippocampus (Zeise et al., 1997; Wang et al., 2000; Viviani et al., 2007; Schafers and Sorkin, 2008). The role of IL-1β in seizure sensitivity was further supported by an *in vitro* system that demonstrated that *N*-methyl-D-aspartate (NMDA)-mediated Ca^2^^+^ influx is enhanced by activation of Src-dependent NMDA receptor subtype 2B (NR2B) phosphorylation (Viviani et al., 2003). In addition to PAMPs, DAMPs have been recently shown to stimulate TLRs, especially TLR4, to exacerbate seizures. The endogenous signals of DAMPs, such as high mobility group box 1 (HMGB1), can be released by stressed neurons or activated microglia and astrocytes, suggesting that LPS may have mimicked the endogenous DAMP pathway to cause seizure (Maroso et al., 2010). Astrocytes can also enhance seizure induction through increased release of glutamate when the IL-1R/TLR pathway is activated (Wetherington et al., 2008; Friedman et al., 2009).

After the activation of the IL-1R/TLR pathway in the glial population, upregulation of COX-2 and prostaglandins in neurons often ensues (Yamagata et al., 1993; Rozovsky et al., 1994; Yoshikawa et al., 2006; Kulkarni and Dhir, 2009). Inhibition of COX-2 activation prior to seizure induction results in increased mortality and exacerbated seizures behaviors in mice (Baik et al., 1999; Toscano et al., 2008). In contrast, ablation of COX-2 production after seizure induction has been shown to be neuroprotective and resulted in decreased production of inflammatory cytokines by glia and prevented leakage of BBB in a conditional COX-2 knockout mouse strain (Serrano et al., 2011). However, constitutive inhibition of COX-2 failed to prevent the recurrent of unprovoked seizures (Holtman et al., 2010). Thus, it remains unclear whether induction of COX-2 in neurons leads to enhanced epileptogenesis.

After the induction of status epilepticus, serum albumin is detected in the brain suggesting that in BBB failure may contribute to the development of epilepsy (van Vliet et al., 2007). Extravasation of albumin into the cerebral cortex as a result of compromised BBB leads to activation of the TGF-β signaling pathway in astrocytes, and hence increases local inflammation. Such inflammatory responses in the brain parenchyma would most likely induce another wave of neuronal hyperactivation and attrition, which leads to excretion of danger signals, such as DAMPs that would further activate glia to boost inflammation. Thus, a positive feedback loop involves seizure, glia, neurons, and immune responses in the brain.

### INFILTRATING IMMUNE MEDIATORS OF THE BRAIN

It has long been appreciated that prolonged seizures lead to upregulation of adhesion molecules on brain endothelial cells to facilitate extravasation of circulating leukocytes. Expression of E-selectin, P-selectin, intracellular adhesion molecule-1 (ICAM-1), and vascular cell adhesion molecule-1 (VCAM-1) are increased on the endothelial cells of the brain (Bell and Perry, 1995; Librizzi et al., 2007). The ligands of these molecules, integrins and mucins, are expressed by circulating leukocytes after seizure and facilitate rolling and tethering of granulocytes and lymphocytes (Fabene et al., 2008). Blockade of α4β1 integrins on leukocytes inhibits infiltration of this cell population into the brain, and therapeutic inhibition of α4 integrin activation prevents induction of seizure, and even development of epilepsy (Fabene et al., 2008).

## CLINICAL EVIDENCE OF THE INVOLVEMENT OF IMMUNE RESPONSES IN EPILEPSY

### ANTI-INFLAMMATORY THERAPIES THAT ARE EFFECTIVE AT TREATING EPILEPSY

The efficacy of anti-inflammatory medications, such as corticosteroids and adrenocorticotrophic hormone (ACTH), in the treatment of some pediatric epilepsies that do not respond to conventional anticonvulsants was one of the first lines of clinical evidence that epilepsy has an immune inflammatory component (Hrachovy et al., 1983; Mackay et al., 2004). It has been shown that ACTH had superior efficacy in the cessation of spasms, improved developmental prognosis, and normalization of electroencephalography (EEG; Snead et al., 1983; Baram et al., 1996; Kivity et al., 2004). In the treatment of refractory epileptic encephalopathies, such as West syndrome, Ohtahara syndrome, Dravet syndrome, Lennox-Gastaut syndrome, Landau-Kleffner syndrome, epilepsy with continuous spike waves during slow-wave sleep and drug-resistant myoclonic atonic epilepsy, a significant proportion of ACTH- and steroid-treated pediatric patients were reported to be seizure-free for an extended period of time, albeit relapsed over time (Yamatogi et al., 1979; Snead et al., 1983; Donat, 1992; Engel, 2001).

In addition to, the corticosteroid therapies, intravenous gammaglobulin (IVIG) has been considered as another potential treatment for refractory epilepsy (Eibl and Wedgwood, 1989). The mechanisms of IVIG induced immunomodulation include suppression of proinflammatory cytokines, interference with antibody-dependent cytotoxicity through Fc receptor blockade, dampening innate immune responses by inhibition of phagocytosis by antigen presenting cells and complement uptake, as well as neutralization of autoantibodies. IgG has been reported to be readily detected in the cerebrospinal fluid (CSF) after a single dose of IVIG in neuromuscular disorders, suggesting that IVIG is capable of crossing the BBB (Cutler et al., 1970; Sekul et al., 1994; Dalakas, 1998). Furthermore, the compromised BBB in many types of epilepsy might further facilitate delivery of IVIG into the brain to exert local immuno- and neuro-modulating effects for seizure alleviation. In a double-blind clinical trial, seven doses of IVIG was administered over a time period of 6 weeks, and more than 50% of the patients in the treatment group had a significant reduction of seizures (van Rijckevorsel-Harmant et al., 1994). Similar effects of seizure reduction and temporary EEG normalization were observed in another trial using pediatric patients (Hart et al., 1994). The use of IVIG in intractable epilepsy and status epilepticus merits further investigation as consistent efficacious outcome has not been achieved and the dosing regimen remains to be optimized.

Blockade of cell-adhesion molecules involved in lymphocyte trafficking has also shown promise in ameliorating seizure severity in epilepsy. Natalizumab, an FDA approved humanized antibody specific to a homing molecule (α4β1 integrin) that directs lymphocyte migration to inflamed tissues, including the brain, has been shown to significantly reduce generalized seizures and status epilepticus in adult patients who also suffered from a autoimmune demyelinating disease, multiple sclerosis (Ley et al., 2007; Sotgiu et al., 2010; Fabene et al., 2013)

### EPILEPTOGENIC TRIGGER WITH AN INTRINSIC INFLAMMATORY NATURE

Febrile status epilepticus is intrinsically associated with immune responses. Genetic susceptibility to inflammation, though not an obligatory factor, has been suggested to lower the seizure threshold, as nearly 30% of febrile seizure patients have such a family history. In addition, mutations in the IL-1β gene segment predispose patients to prolonged febrile convulsions (Millichap, 1959; Virta et al., 2002; Kanemoto et al., 2003). An elevation in a number of proinflammatory cytokines caused by neurotropic viral infections, for instance, human herpesvirus-6 and influenza viruses, is also commonly associated with febrile seizures in infants and young children (Hall et al., 1994; Chiu et al., 2001). Detection of viral DNA is more frequent in the CSF of patients with repetitive febrile seizures than in those patients with a single seizure (Kondo et al., 1993). Increased levels of Th1 and Th2 cytokines, such as interferon-γ (IFN-γ) and interleukin-6 (IL-6), have been reported in influenza-infected patients who later developed febrile seizures, when compared with the virally infected control subjects without seizures (Chiu et al., 2001; Masuyama et al., 2002; Kawada et al., 2003).

Rasmussen’s encephalitis, a prototypic childhood inflammatory epilepsy, is a progressive immune-mediated brain disorder characterized by focal recurrent seizures (epilepsia partialis continua), unilateral hemispheric atrophy, progressive neurological dysfunction and intractable epilepsy. Rasmussen encephalitis is associated with T-cell activation and production of proinflammatory cytokines by activated glia. Effector CD8^+^ cytotoxic T lymphocytes are proposed to induce astrocytic and neuronal apoptosis and degeneration, one of the hallmarks of Rasmussen’s encephalitis (Bauer et al., 2007). An autoantigen, a glutamate receptor, GluR3, has been detected in this disease. Removal of the GluR3-specific antibody from the circulation has been shown to ameliorate seizure severity, promote neurological functions, and improve the disease prognosis. However, anti-GluR3 antibody is not present in most cases and immune modulation including IVIG, steroid and plasmapheresis has limited efficacy (Rogers et al., 1994; He et al., 1998; Mantegazza et al., 2002; Watson et al., 2004). Hemispherectomy remains the only “cure” of disease progression.

In recent years, an increasing number of autoantibodies have been detected in serum and CSF of patients with new onset drug-resistant focal epilepsy. A definitive diagnosis and therapy have thus been possible for cases that would have been previously categorized as viral or idiopathic encephalitis/epilepsies. The California Encephalitis Project found that the frequency of autoimmune encephalitis was greater than any single viral etiology. Anti-*N*-methyl D-aspartate receptor (NMDAR) encephalitis was, in fact, the most frequent cause of immune-mediated encephalitis (Armangue et al., 2013). Autoimmune/inflammatory epilepsy is defined as immunologically mediated disorder where recurrent seizures are prominent feature and immune etiology is suggested by detection of neuronal antibodies, presence of inflammatory changes in CSF or upon MRI, or immunotherapy-responsive symptoms and exclusion of other etiologies. Autoimmune/inflammatory epilepsies include limbic encephalitis (both paraneoplastic and non-paraneoplastic), non-limbic encephalitis complicated by seizures, seizures in the context of autoimmune disease, and neural antibody-mediated CNS disorders where seizures are a significant feature. Disease causing autoantibodies have been detected against the following specific neuronal surface proteins: NMDAR, α-amino-3-hydroxy-5-methyl-4-isoxazolepropionic acid receptor (AMPAR), γ-aminobutyric acid beta receptor (GABA-BR), glycine receptor, voltage gated potassium channel leucine-rich glioma-inactivated 1 (VGKC LGI1), VGKC CASPR2 and P/Q or N-type voltage-gated calcium channel (VGCC; Armangue et al., 2012).

The paraneoplastic type is thought to be caused by self-reactive T lymphocytes, although antibodies specific for intracellular components are often detectable (Dalmau and Rosenfeld, 2008). The non-paraneoplastic category is consistently associated with seizure-inducing autoantibodies that target extracellular membrane components, including voltage-gated channels, NMDAR, and glutamic acid decarboxylase (Bien et al., 2007; Dalmau et al., 2008; Vincent and Bien, 2008; Dalmau, 2009; Vincent et al., 2010). These antibodies cause seizures by modifying neuronal excitability, which has been recapitulated in an *in vitro* model by measuring hippocampal neuronal firing frequency following autoantibody treatment (Vianello et al., 2008). Due to its autoimmune nature, limbic encephalitis responds to immunotherapies, including steroids and IVIG through the mechanisms of dampening T-cell immunity and neutralizing autoantibodies (Dalmau, 2009). Another type of autoimmune epilepsy that also responds to corticosteroid treatments is Hashimoto’s encephalopathy. Self-reactive antibodies in this syndrome target thyroid peroxidase or thyroglobulin, and are very potent in seizure induction (Castillo et al., 2006; Watemberg et al., 2006).

### DETECTION OF IMMUNE MEDIATORS IN THE BRAIN OF PATIENTS WITH EPILEPSY

Leukocyte accumulation in the perivascular space, and occasionally the parenchyma, of epileptic brain in pediatric as well as adult patients provide another line of evidence that immune cell invasion of the central nervous system (CNS) may be critical in orchestrating epileptogenesis. CD3^+^ lymphocytes and myeloid-derived macrophages have been detected in resected brain samples of patients diagnosed with temporal lobe epilepsy (TLE; Ravizza et al., 2008). A subset of CD3^+^ cells, cytotoxic CD8^+^ T lymphocytes, has also been found in the gray and white matter of epileptic patients with tuberous sclerosis complex (Boer et al., 2008). Brain-infiltrating granulocytes and T cells have been additionally demonstrated in epilepsy of diverse etiologies (Fabene et al., 2008). In line with the above findings, our laboratory and others have detected a myriad of immune-related genes that are upregulated in surgical specimens from patients with intractable TLE using a global survey of changes in gene expression. These immune-related genes include chemokines, complement components, metalloproteinases and their inhibitors, adhesion molecules, immune receptors such as MHC molecules and Fc receptors, and heat shock proteins (Aronica and Gorter, 2007; van Gassen et al., 2008).

## PROPOSED IMMUNE MECHANISMS OF EPILEPTOGENESIS

Understanding the immune mechanisms underlying epileptogenesis provide insights into the development of more effective target-specific immunotherapies rather than general treatments that non-specifically suppress or regulate the immune responses. Ample experimental and clinical evidence has suggested that inflammation in the brain is likely to predispose, precipitate, and perpetuate epileptogenesis, however, protective immune responses that promote neuronal repair and restores homeostasis also exist in epilepsy as well as other immune-mediated neurological disorders, such as multiple sclerosis, Parkinson’s disease, and Alzheimer’s neurodegeneration. Thus, whether the immune response detected during the initiation and development of epilepsy is always deleterious to the survival of brain cells or perhaps may also mediate neuroprotective functions merits further in-depth investigation.

The current dominant view of immune-mediated epileptogenesis entails contributions from both brain-resident cells capable of innate immune responses as well as peripherally derived infiltrating innate and adaptive immune effector cells. The pathological triggering events that are initiated in the brain or the periphery for a variety of reasons, such as simple febrile seizures, trauma, stroke or infection, may lead to an inflammatory cascade. Activation of glia, neurons, and endothelial cells that constitute the BBB most likely result in the release proinflammatory cytokines, such as IL-1β and TNF-α, and danger signals, such as HMGB1.

These factors activate cognate pathways in neurons to cause an intracellular calcium ion surge, which results in modification of voltage-dependent ion channels. Dysregulated ion channels directly enhance the neuronal hyperexcitability and reduce seizure threshold. In addition, proinflammatory cytokines also stimulate chronic release of neuroexcitatory transmitters, inhibit uptake of these neurotransmitters by the glial population, and restrict the recycling of GABA receptors (Hu et al., 2000; Bezzi et al., 2001; Stellwagen et al., 2005; Ferguson et al., 2008). COX-2 and prostaglandin can also be involved in such a process that remodels the neuronal network by mobilizing intracellular calcium storage and an increase in cAMP production.

The inflammatory milieu and the neuronal hypersynochronization in the CNS are often accompanied by BBB leakage, which introduces tightly regulated blood components, such as albumin and potassium ion, into the brain (Seiffert et al., 2004; Oby and Janigro, 2006; Aronica et al., 2007; Ivens et al., 2007; Shlosberg et al., 2010). Increased leukocyte adhesion to the endothelial cells further modifies the BBB through cytoskeletal organization, which results in enhanced leukocyte infiltration into the brain (Greenwood et al., 2002). Upon entering the brain, activated peripheral immune cells are capable of generating free radicals, releasing additional chemokines, cytokines, nitric oxide, and cytotoxic enzymes to establish a self-amplifying cascade to further precipitate epileptogenesis.

## CONCLUSION

**FIGURE 1:**
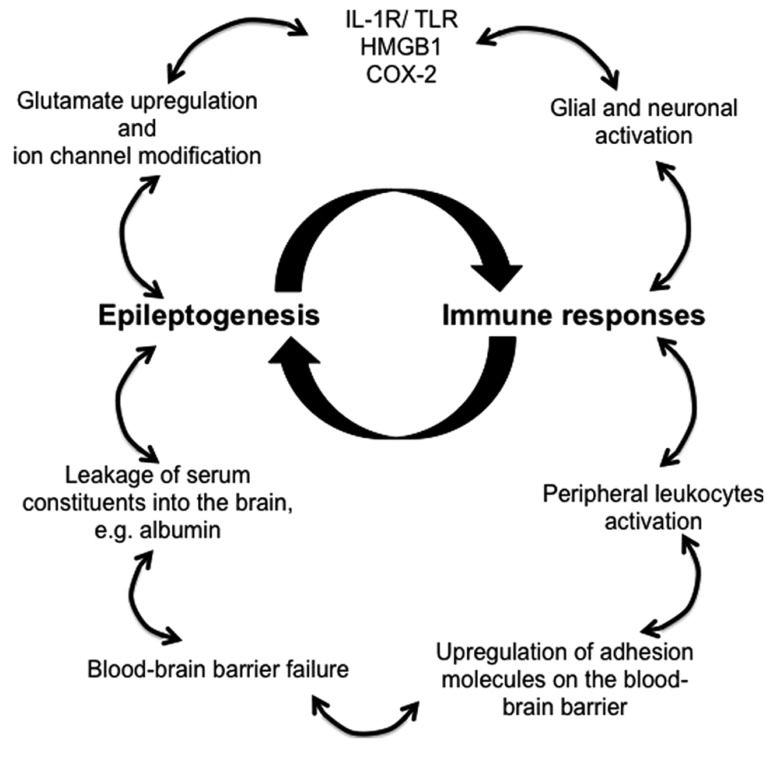
**Proposed immune mechanisms in epileptogenesis**. The relationship between the immune system and the development of epilepsy is non-linear, but rather better represented as an amplifying feedback loop. The immune response predisposes, precipitates and perpetuates epileptogenesis through activation of resident brain cells (glia and neurons) and facilitation of peripheral leukocyte infiltration. These immune mediators release proinflammatory cytokines and chemokines into the brain parenchyma and the blood, thereby activating downstream signaling cascades and compromising blood–brain barrier, which leads to pathophysiological outcomes, reoccurrence of seizures, and ultimately the development of epilepsy.

Animal models and clinical evidence highlight the involvement of CNS resident and peripherally derived infiltrating immune mediators in seizure induction and epilepsy development (Vezzani et al., 2011a). Robust immune responses in the brain decrease seizure threshold, enhance neuronal excitability, induce BBB failure, promote synaptic reorganization, and regulate epileptogenesis (**Figure 1**). Despite the appreciation of the critical role of immunity in epileptogenesis and the advancements made in the recent years in understanding the immunological mechanisms underlying epilepsy, novel diagnostic measures and effective therapeutic treatments that targets immunological pathways are still lacking.

In addition to, the glial populations and neurons, which are the major brain-resident immune mediators in epilepsy, peripheral leukocytes that infiltrate the brain are also being investigated for their contribution to epileptogenesis as a result of a compromised BBB, for instance, macrophages, monocytes, dendritic cells, αβ T lymphocytes, γδ T lymphocytes, and regulatory cells. Several inflammatory signaling pathways have been identified which initiate immune responses involving the aforementioned immune mediators. Activation of the IL-1R/TLR pathway may be due to brain injury or infection, but could also be caused by DAMPs, such as HMGB1. Furthermore, COX-2 induced production of prostaglandins is capable of triggering brain inflammation (Fabene et al., 2008; Serrano et al., 2011). Therefore, pharmacological blockade of these signaling pathways and inhibitors that antagonizing the main immune mediators have the potential of becoming the next generation of effective anti-epileptic treatment.

## Conflict of Interest Statement

The authors declare that the research was conducted in the absence of any commercial or financial relationships that could be construed as a potential conflict of interest.

## Abbreviations

ACTH: adrenocorticotrophic hormone
AMPAR: α-amino-3-hydroxy-5-methyl-4-isoxazolepropionic acid receptor
BBB: blood–brain barrier
CNS: central nervous system
COX-2: cyclooxygenase-2
CSF: cerebrospinal fluid
DAMPs: damage-associated molecular patterns
EEG: electroencephalography
FDA: U.S. food and drug administration
GABA: gamma-aminobutyric acid
GABA-BR: gamma-aminobutyric acid beta receptor
GLuR3: glutamate receptor 3
HMGB1: high mobility group box 1
ICAM-1: intracellular adhesion molecule-1
IFN-γ: interferon-γ
IgG: immunoglobulin G
IL-1β: interleukin-1β
IL-1R/TLR: interleukin-1 receptor/toll-like receptor
IL-6: interleukin-6
IVIG: intravenous gammaglobulin
LPS: lipopolysaccharide
NMDA: *N*-methyl-D-aspartate
NMDAR: *N*-methyl D-aspartate receptor
NR2B: NMDA receptor subtype 2B
PAMPs: pathogen-associated molecular patterns
Smad: small mothers against decapentaplegic
TGF-β: transforming growth factor-β
TLE: temporal lobe epilepsy
TNF-α: tumor necrosis factor-α
VCAM-1: vascular cell adhesion molecule-1
VGCC: N-type voltage-gated calcium channel
VGKC LGI1: glycine receptor, voltage gated potassium channel leucine-rich glioma-inactivated 1.
